# Atrial fibrillation is a predictor of nonobstructive coronary artery disease in elective angiography in old age: a cross-sectional study in Poland and Russia

**DOI:** 10.1007/s40520-021-01895-y

**Published:** 2021-06-11

**Authors:** Ewelina Rogalska, Łukasz Kuźma, Zyta B. Wojszel, Anna Kurasz, Dmitry Napalkov, Anastasiya Sokolova, Anna Tomaszuk-Kazberuk

**Affiliations:** 1 Department of Cardiology, Medical University of Bialystok, M. Sklodowska-Curie Str. 24A, 15-276 Bialystok, Poland; 2 Department of Invasive Cardiology, Medical University of Bialystok, M. Sklodowska-Curie Str. 24A, 15-276 Bialystok, Poland; 3 Department of Geriatrics, Medical University of Bialystok, Fabryczna Str. 27, 15-369 Bialystok, Poland; 4 Department of Internal Diseases, I.M. Sechenov First Moscow State Medical University, Bol’shaya Pirogovskaya Ulitsa, 19c1, Moscow, Russia 119146

**Keywords:** Elective coronary angiography, Nonobstructive coronary lesions, Older patients, Atrial fibrillation, Chronic coronary disease

## Abstract

**Background:**

Significant changes in the coronary vessels are not confirmed in a large proportion of patients undergoing cardiac catheterization.

**Aims:**

The present study aimed to determine correlates and independent predictors of nonobstructive coronary artery disease (CAD) in older adults referred for elective coronary angiography.

**Methods:**

A cross-sectional study was conducted involving 2,214 patients referred to two medical centers (in Poland and Russia) between 2014 and 2016 for elective coronary angiography due to exacerbated angina, despite undergoing optimal therapy for CAD. The median age was 72 years (IQR: 68–76), and 49.5% patients were women.

**Results:**

Significant stenosis (defined as stenosis of 50% or more of the diameter of the left main coronary artery stem or stenosis of 70% or more of the diameter of the remaining major epicardial vessels) was diagnosed only in 1135 (51.3%) patients. Female sex (odds ratio [OR], 3.01; 95% confidence interval [CI], 2.44–3.72; *p* < 0.001) and atrial fibrillation (OR, 1.87; 95% CI 1.45–2.40; *p* < 0.001) were the main independent predictors of nonobstructive CAD. Significantly lower ORs were observed for diabetes (OR, 0.75; 95% CI 0.59–0.95; *p* = 0.02), chronic kidney disease (OR, 0.76; 95% CI 0.61–0.96; *p* = 0.02), and anemia (OR, 0.69; 95% CI 0.50–0.95; *p* = 0.02) after controlling for age, chronic heart failure, BMI, and study center.

**Discussion and conclusions:**

The results confirmed that nonobstructive CAD occurs in a high percentage of older patients referred for elective coronary angiography. This suggests the need to improve patient stratification for invasive diagnosis of CAD, especially for older women and patients with atrial fibrillation.

**Trial registration number and date of registration:** NCT04537507, September 3, 2020.

## Introduction

The older population is at an increased risk of coronary artery disease (CAD), the leading cause of morbidity and mortality in developed countries [1]. Although some positive trends are observed in this respect due to declining physical inactivity and smoking [2], the number of patients with CAD will continue to remain high due to demographic aging and the epidemic of obesity and diabetes [3, 4]. This will contribute to the increasing need to undertake therapeutic decisions regarding acute coronary syndromes (ACSs) and stable coronary disease in older patients [5].

From the results of previous research, it is clear that a strategy of early invasive management and revascularization in patients with ACSs provides substantial benefits, even in frail older adults [6, 7]. The results of more recent studies indicate that in addition to improving exercise tolerance and quality of life, coronary artery bypass grafting (CABG) and percutaneous revascularization (PCI) using new-generation drug-eluting stents may also have a positive effect on the prognosis of older patients with chronic CAD [8]. Cost-effectiveness analysis results indicate that, from a long-term perspective, PCI treatment of elderly patients can reduce the expenditure [9].

Because of the increased risk of complications associated with coronary angiography in old age [10–12], the qualification to undergo this procedure must be as precise as possible to avoid overtreatment. The “geriatric approach” should also be considered, as with the advancement of age, the priority becomes the quality of life, life capability, the lack of persistent clinical symptoms of CAD, or even the possibility of reducing the number of medications taken and the risk of drug interactions [13]. Nevertheless, we should consider that it is also sometimes necessary to have “negative” diagnoses. Not having undergone coronary angiography could imply that a patient would be indefinitely considered as a “coronary” patient. Accordingly, this will result in the maintenance of their antianginal or antiplatelet drugs without any clinical benefits and, perhaps, with an increased incidence of adverse events. In patients without any apparent cardiovascular disease, antiplatelet therapy is not recommended for primary prevention due to the high risk of bleeding complications. On the other hand, the exclusion of significant coronary atherosclerotic lesions (e.g., angiographically insignificant lesions, microvascular disease) does not always entitle us to withdraw acetylsalicylic acid (ASA). Additionally, these patients are often burdened with atherosclerosis, which increases the likelihood of neurological episodes such as transient ischemic attack (TIA) or stroke. Therefore, referring patients for coronary angiography to rule out ischemic heart disease to discontinue these drugs is not necessarily a good strategy.

In recent years, there has been a growing concern of high rates of nonobstructive CAD identified during elective coronary angiography [14]. This indicates that better strategies for risk stratification in stable angina are needed to make informed decisions and to increase cardiac catheterization diagnostic yield. Nevertheless, little is known about the effectiveness of coronary angiography in CAD and the determinants of nonobstructive CAD in older adults.

Therefore, in the present study, we aimed to analyze which factors are correlates and independent predictors of the lack of significant coronary angiography lesions in older patients referred for elective procedure and whether atrial fibrillation (AF), the most common arrhythmia in elderly patients, affects the outcome of cardiac catheterization.

## Materials and methods

### Participants and study design

We conducted a retrospective cross-sectional study in patients above 65 years of age who were referred for elective coronary angiography between 2014 and 2016 to the Department of Invasive Cardiology of the Medical University of Bialystok, Bialystok, Poland (MUB), and I.M. Sechenov First Moscow State Medical University, Moscow, Russian Federation (MSMU). This is a subanalysis of broader multicenter observational trial results [15].

**Fig. 1:**
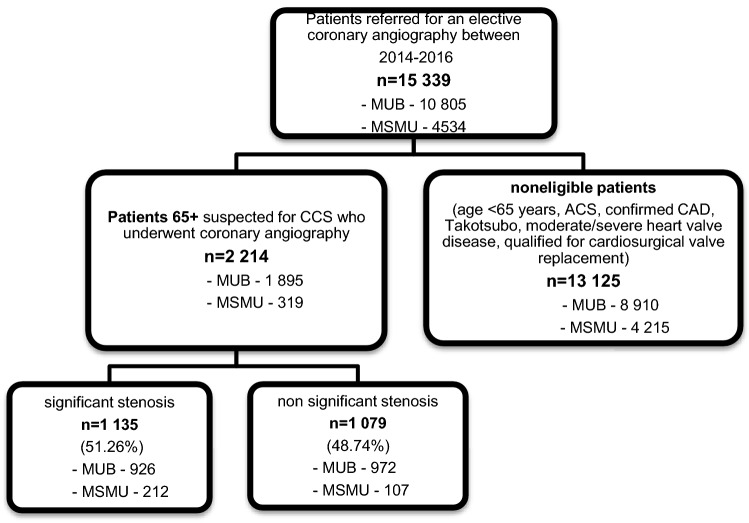
Flow chart of patient enrolment. *ACS* acute coronary syndrome, *CAD* coronary artery disease, *CCS* chronic coronary disease, *MSMU* I.M. Sechenov First Moscow State Medical University, Moscow, Russian Federation, *MUB* Medical University of Bialystok, Poland

We included all consecutive patients referred for coronary angiography due to exacerbated angina (recurrent chest pain, classical stable angina, long history of chest pain/angina, or other symptoms such as dyspnea), despite undergoing optimal therapy of CAD under the prevailing recommendations. We excluded patients with ACSs, Takotsubo cardiomyopathy, a history of earlier confirmed ischemic heart disease and a prior diagnosis of moderate or severe heart valve disease and those qualified for cardiosurgical valve replacement (Fig. 1). Although we do not have data on the medications taken by the patients, the treatment was based on the European Society of Cardiology (ESC) guidelines for AF and chronic coronary syndrome (CCS) at the time of hospitalization.

### Study parameters

We retrieved all variables that characterized patient details from their medical charts. Information on age, gender, prevalence of diseases associated with increased risk of CAD (chronic cardiac failure, hypertension, AF, diabetes, hyperlipidemia, liver failure, and anemia), and results of coronary angiogram was collected. The diagnosis of CCS and indication for PCI were established according to the existing European guidelines [16]. Significant stenosis of the coronary vessel was defined as stenosis of 50% or more of the diameter of the left main coronary artery stem or stenosis of 70% or more of the diameter of the remaining major epicardial vessels (“stenosis + ” cases). Those patients who did not meet this criterion were classified as “stenosis − ” cases. We also assessed the localization of significant stenosis in the coronary arteries and classified the coronary artery disease as single- or multivessel disease based on the localization.

Left ventricular ejection fraction (LVEF) was assessed by routine transthoracic echocardiography using the modified biplane Simpson’s method, following the recommendations of the European Society of Echocardiography [17]. AF was diagnosed on the basis of medical history, 24-h Holter monitoring, and ECG on admission. AF was subclassified into paroxysmal AF or chronic AF—persistent and permanent [18].

Chronic kidney disease (CKD) was diagnosed according to the KDIGO 2012 Clinical Practice Guideline for the Evaluation and Management of Chronic Kidney Disease [19]. CKD was defined as the presence of kidney damage or an estimated glomerular filtration rate (eGFR) lower than 60 ml/min/1.73 m^2^, persisting for at least 3 months. We determined eGFR using the CKD-EPI formula. The nutritional health was evaluated based on body mass index (BMI), and the patient was classified as obese if BMI was ≥ 30 kg/m^2^. Diabetes was considered based on the patient’s history or the use of antidiabetic treatment. A history of systolic blood pressure of ≥ 140 mmHg, a diastolic pressure of ≧90 mmHg, or the use of antihypertensive drugs for treatment was considered for hypertension. A history of hyperlipidemia or the use of antihyperlipidemic drugs was considered for hyperlipidemia. Anemia was defined as hemoglobin < 13 g/dL (8.07 mmol/L) for men and < 12 g/dL (7.45 mmol/L) for women (measured on admission). Liver failure was diagnosed if there was a history of cirrhosis, bilirubin levels above twice the upper limit of normal, or transaminase or alkaline phosphatase levels above three times the upper limit.

### Statistical analysis

Data were collected and analyzed by IBM SPSS Version 18 Software package (SPSS, Chicago, IL, USA). We used the Kolmogorov–Smirnov test to assess the distribution of variables. As all continuous variables were not normally distributed, we presented them as median and interquartile range. Categorical variables were presented as the number of cases and percentage. The Mann–Whitney *U* test and the *χ*^2^ test were used to determine the statistical significance of differences in independent variables—chosen based on the literature and available in the database—between “stenosis + ” and “stenosis − ” cases. A multivariable logistic regression was performed after bivariate analyses. It included all predictors with a P value of less than 0.1 and without a significant multicollinearity effect. The variance inflation factor was used to identify a correlation—and the strength of that correlation—between independent variables. The differences were considered to be significant at a two-tailed P value of less than 0.05.

## Results

A total of 2214 patients (1895 in MUB and 319 in MSMU) fulfilled the inclusion criteria of the analysis. The median age of patients was 72 (68–76) years. More than one-third of the patients were above 75 years of age (37.0%), and almost half of them were female (49.5%). Significant stenosis in coronary angiography was diagnosed in 1135 (51.3%) patients. In most cases (*n* = 660; 58.1%), the diagnosis was a multivessel CAD. Significant stenosis was observed most often in the left anterior descending artery (*n* = 737; 64.9%) and the right coronary artery (572; 50.4%), and less often in the circumflex artery (408; 35.9%), the diagonal artery (261; 23%), the left marginal artery (242; 21.3%), and the left main artery (113; 10%).

Table 1 shows the characteristics of the study groups. The “stenosis − ” cases were significantly younger (median age 72 years; IQR, 68–76 vs. 73; IQR 68–77 in “stenosis + ” group, *p* = 0.03) and predominantly female (61.6% vs. 39.3%, *p* < 0.001). These patients showed significantly less often diabetes (24.7% vs. 28.9%, *p* = 0.03) and CKD (31.2% vs. 37.5%, *p* < 0.001), but significantly more often AF (31.4% vs. 25.2%, *p* < 0.001). They had significantly higher BMI (28.7 kg/m^2^; IQR 26.0–32.0 vs. 28.4 kg/m^2^; IQR 25.6–31.2, *p* = 0.02), but no differences were observed in the prevalence of obesity, hypertension, chronic heart failure, hyperlipidemia, anemia, liver failure, or LVEF. The nonobstructive CAD was significantly more frequent in the patients from the Polish center (51.3% vs. 33.5%, *p* < 0.001). Vascular complications following coronary angiography were reported in 0.5% of patients (n = 12), while neurological complications occurred in 0.13% (*n* = 3), with no significant differences between the groups. Regarding the reduction in kidney function, post-contrast acute kidney injury occurred in 2.82% (*n* = 32) of “stenosis + ” patients and 1.39% (*n* = 15) of “stenosis − ” patients (*p* < 0.001). Additionally, one in-hospital death was reported in the “stenosis + ” group. The “stenosis + ” group had a higher CHA_2_DS_2_-VASc score (mea* n* = 3.90, SD = 1.38; Me = 4, IQR 3–5) than the “stenosis − ” group (mea* n* = 3.29, SD = 1.12; Me = 3, IQR 3–4).

**Table 1:** Patient characteristics (*n* (%) or median [IQR])

	Total (*n* = 2214)	Stenosis + (*n* = 1135)	Stenosis − (*n* = 1079)	*p* value
Age, *years*	72.0 [68.0–76.0]	73.0 [68.0–77.0]	72.0 [68.0–76.0]	0.03
Age 75 + years	819 (37.0)	455 (40.1)	364 (33.7)	0.002
Female	1095 (49.5)	430 (39.3)	665 (61.6)	< 0.001
Study center				
MUB	1895 (85.6)	923 (48.7)	972 (51.3)	< 0.001
MSMU	319 (14.4)	212 (66.5)	107 (33.5)	< 0.001
BMI, *kg/m*^*2*^	28.5 [25.8–31.6] (*n* = 1657)	28. 4 [25.6–31.2] (*n* = 811)	28.7 [26.0–32.0] (*n* = 846)	0.02
Obesity (BMI > 30 kg/m^2^)	612 (36.9)	282 (34.8)	330 (39.0)	0.08
Hypertension	1950 (88.1)	1002 (88.3)	948 (87.9)	0.79
Diabetes mellitus	595 (26.9)	328 (28.9)	267 (24.7)	0.03
Hyperlipidemia	1176 (53.1)	617 (54.4)	559 (51.8)	0.23
Chronic heart failure	434 (19.6)	240 (21.1)	194 (18.0)	0.06
LVEF*, %*	55 [45–60] (*n* = 1110)	55 [46–60] (*n* = 627)	55 [45–60] (*n* = 483)	0.96
LVEF < 50%	342 (30.8)	191 (30.5)	151 (31.3)	0.79
AF	625 (28.2)	286 (25.2)	339 (31.4)	< 0.001
Paroxysmal	310 (14.0)	146 (12.9)	164 (15.2)	
Persistent	60 (2.7)	29 (2.6)	31 (2.9)	
Chronic	255 (11.5)	111 (9.8)	144 (13.3)	
Chronic kidney disease	763 (34.5)	426 (37.5)	337 (31.2)	< 0.001
eGFR, *mL/min /1.73 m*^*2*^	70.1 [56.8–81.6]	68.8 [55.2–81.8]	71.2 [58.3–81.6]	0.02
Anemia	275 (12.4)	155 (13.7)	120 (11.1)	0.07
Liver failure	66 (3.0)	29 (2.6)	37 (3.4)	0.23

*AF* atrial fibrillation, *BMI* body mass index, *eGFR* estimated glomerular filtration rate, *IQR* interquartile range, *LVEF* left ventricular ejection fraction, *MSMU* I.M. Sechenov First Moscow State Medical University, Moscow, Russian Federation; MUB, Medical University of Bialystok, Poland; n, number; Stenosis + , patients with significant stenosis in coronary vessels (obstructive CAD); Stenosis − , patients without significant stenosis in coronary vessels (nonobstructive CAD)

**Fig. 2:**
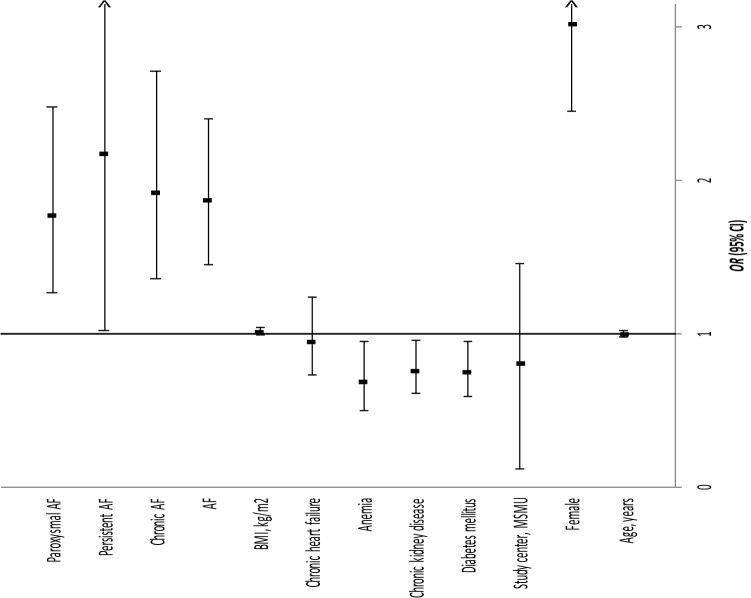
Summary of factors associated with the lack of significant stenosis in the coronary arteries

A direct logistic regression analysis was performed on “stenosis − ” as the outcome and nine predictors: age, sex (female), AF, diabetes, CKD, anemia, chronic heart failure, BMI, and study center (Fig. 2, Table 2, Model 1). Significantly higher odds ratios (ORs) for the absence of significant stenosis were observed for female sex (OR, 3.01; 95% CI 2.44–3.72; *p* < 0.001) and AF (OR, 1.87; 95% CI 1.45–2.39; *p* < 0.001) and significantly lower ORs were observed for diabetes (OR, 0.75; 95% CI 0.59–0.95; *p* = 0.02), CKD (OR, 0.76; 95% CI 0.61–0.96; *p* = 0.02), and anemia (OR, 0.69; 95%CI, 0.50–0.95; *p* = 0.02) after controlling for age, chronic heart failure, BMI, and study center. An overall prediction success rate of 63.5% was observed, with 66.1% correct prediction of the “stenosis − ” status (sensitivity) and 60.9% correct prediction of “stenosis + ” status (specificity).

**Table 2:** Factors associated with the lack of significant stenosis in the coronary arteries – direct multivariable logistic regression models

Variables	OR	95% CI	*P* value	OR	95% CI	*P* value
		Model 1			Model 2	
Age, *years*	0.998	0.98–1.02	0.84	0.998	0.98–1.02	0.84
Female	3.01	2.44–3.72	< 0.001	3.02	2.45–3.73	< 0.001
Study center, *MSMU*	0.81	0.12–5.47	0.83	0.81	0.12–5.46	0.83
Diabetes mellitus	0.75	0.59–0.95	0.02	0.75	0.59–0.95	0.02
Chronic kidney disease	0.76	0.61–0.96	0.02	0.76	0.61–0.96	0.02
Anemia	0.69	0.50–0.95	0.02	0.69	0.50–0.95	0.02
Chronic heart failure	0.96	0.73–1.24	0.73	0.95	0.73–1.24	0.69
BMI, *kg/m*^*2*^	1.01	0.99–1.04	0.26	1.01	0.99–1.04	0.26
AF	1.87	1.45–2.40	< 0.001			
Sinus rhythm				1.0		
Chronic AF				1.92	1.36–2.71	< 0.001
Persistent AF				2.17	1.02–4.64	0.045
Paroxysmal AF				1.77	1.27–2.48	0.001

*AF* atrial fibrillation, *BMI* body mass index, *CI* confidence interval, *MSMU* I.M. Sechenov First Moscow State Medical University, Moscow, Russian Federation; *OR* odds ratio

To test whether the type of AF—paroxysmal, persistent, or chronic AF—was an influencing factor, we constructed Model 2. Apart from sex (female), diabetes, CKD, anemia, chronic heart failure, BMI, and study center, we included three types of AF instead of the variable “AF,” with the variable “sinus rhythm” as the reference category. In the regression analysis, all AF types (notably the chronic and persistent ones) showed increased ORs for having clear vessels (Table 2, Model 2). An overall prediction success rate of 63.3%—similar to that noted for Model 1—was observed, with 66.4% correct prediction of the “stenosis − ” status (sensitivity) and 60.0% correct prediction of “stenosis + ” status (specificity).

## Discussion

Our study proved that in the group of older adults who underwent elective diagnostic coronary angiography, the percentage of patients in whom no clinically significant atherosclerotic lesions were detected was very high, i.e., almost 50% of the patients. This is a major clinical problem as in a considerable percentage of these cases, it is possible that older patients were unnecessarily exposed to invasive testing, with all the risks associated with this procedure. As shown in previous studies, this is a common issue observed in up to 62.4% of cardiac catheterizations in patients with CAD [20].

Because of recent progress and improvement in the safety of intervention techniques, the risks related to PCIs are now almost similar for older and younger adult populations [21]. Nevertheless, specific risks and difficulties remain for the older adults because of the more complex morphology of the coronary vessels, more frequent multivessel disease, calcifications, or comorbidities, resulting in polytherapy and difficulties in pharmacotherapy [22, 23]. A decision to refer older patients for elective coronary angiography still poses a significant challenge for clinicians, which is a multifactorial issue [13].

Earlier analyses identified several determinants related to the nonobstructive CAD, such as younger age, female sex, atypical presentation, low risk in the noninvasive test result, low comorbidity, and lack of major CAD-associated risk factors [20, 24]. We did not find that age per se played a role in predicting cardiac catheterization results in older patients. In the logistic regression analysis, women had more than three times greater likelihood of having nonsignificant stenosis in the coronary vessels, while patients with AF had almost twice as high likelihood. At the same time, diabetes, CKD, and anemia significantly reduced the possibility of the negative result of elective coronarography.

One of the reasons for female sex being the leading independent predictor of having nonobstructive CAD might be the lower sensitivity and specificity of noninvasive tests in women [25, 26]. More frequently, compared to elderly males, elderly females have less typical chest pain or lack of chest pain in ACSs [27]. While the association with diabetes and CKD seems to be fairly obvious, as these diseases promote coronary atherosclerosis development [28, 29], it is not entirely clear in the case of anemia—a common comorbidity of chronic cardiac failure and CKD in patients undergoing PCIs, resulting in more cardiac complications [30]. Symptomatic CAD patients take ASA, which is associated with the possibility of bleeding—often subclinical—and may result in anemia. Hyperkinetic circulation in persistent anemia may aggravate arterial wall damage, leading to atherosclerosis progression [31]. On the other hand, mild anemia could be a possible cause of false-positive stress echocardiography in nonobstructive CAD, which would indicate the possibility of its opposite effect [32]. In addition to anemia reducing the probability of the lack of significant findings in coronary angiography, the prevalence of anemia was higher in the stenosis + group than in the stenosis − group. However, this difference did not reach statistical significance.

The prevalence of AF—the second main determinant of nonobstructive CAD in our research—was relatively high in the studied group (28.2%). In several studies, AF was associated with the lack of significant coronary lesions in coronary angiography [33, 34]. The prevalence of CAD in patients with AF reaches up to 46% [34], as both diseases share several common risk factors such as hypertension, diabetes, and obesity [35, 36]. As some of the symptoms of AF and CAD overlap, such as dyspnea or chest pain, a significant number of patients are referred for CAD diagnosis [37]. ST-segment depression during rapid AF cannot be solely considered as a factor indicative of underlying ischemia or as a positive stress test equivalent [38]. The same applies to the elevated levels of cardiac troponins in patients with acute symptomatic AF [39].

The decision to conduct cardiac catheterization after noninvasive test results indicate a high risk of significant coronary vessel changes improves the effectiveness of coronary angiography [14, 20]. However, stress testing before the intervention is significantly less often performed in the older age group, and older patients less often show objective signs of ischemia [40].The traditional stress test is often inconclusive in AF, and the frailty of patients with AF is an additional obstacle that limits the possibility of performing stress tests [38, 41]. Because of the possibility of triggering an episode of AF, dobutamine stress ECHO is not considered as the primary alternative [42]. Moreover, tests that could have a higher diagnostic value in older people, such as coronary computed tomography angiography or cardiac single-photon emission computed tomography, have limited accuracy to detect AF [43]. Our study results seem to confirm that the golden method of noninvasive CAD diagnostics in the group of elderly patients with coexisting AF is still being sought.

A significant difference in the frequency of negative coronary angiography findings between the two centers participating in the study might result from differences in eligibility criteria for the coronary angiography procedure between the centers, the availability of the procedure, and the difference in assessing atherosclerotic lesions during coronary angiography. Similar differences between the centers were observed in other studies [14, 20]. Nevertheless, the variable “study center” ultimately did not significantly affect the results of logistic regression.

The main strength of our study is that it was performed using a large clinical dataset, which represented real-life daily clinical practice in two medical centers from two different countries. Nevertheless, the present study has several limitations. First, the study was retrospective in nature. Consequently, we had limited access to certain information such as EF, natriuretic peptide levels, BMI, and smoking history (not available completely for MSMU). In all cases where PCI was possible, the patients received such treatment; however, the treatment data for the remaining patients were incomplete. Furthermore, even though retrospective chart databases provide convenient and cheap access to the data of many patients, potential selection bias should still be considered. The assessment of the functional significance of stenosis relied primarily on the visual assessment of the clinician performing angiography. In our study, the clinicians relatively rarely used fractional flow reserve (FFR) for this purpose; this could result in a more significant stenosis estimation error margin [44]. This variability could have affected our results. Moreover, we did not investigate the effect of myocardial ischemia induced by coronary microvascular disease. Regarding the coexisting diseases, we relied on the diagnoses established by the physicians in charge, and we did not verify them again. Finally, the assessment of independent variables preceded the evaluation of the dependent variable on the timeline (suggesting a prospective nature of the study). Yet, the research we performed was a cross-sectional one. Hence, we can say that the study identified potential factors related to a negative coronary examination result and not its actual “predictors.” The term “predictor” should be considered as a mathematical concept used in regression analysis and not as a determining factor of a phenomenon’s occurrence.

## Conclusions

The results of the present study reflect difficulties in decision-making on qualifying elderly patients for coronary angiography. Almost 50% of older patients referred for elective cardiac catheterization due to CAD had no significant atherosclerotic lesions in the coronary arteries. Female sex and AF were the main factors that increased the odds of nonobstructive CAD, whereas the co-existence of diabetes, CKD, and anemia increased the diagnostic yield of elective coronary angiography. Therefore, qualifying older females with AF and no other major risk factors in particular for invasive diagnostics requires consideration of potential benefits and risks.

## Data Availability

The data supporting the current study results are available from the corresponding author on reasonable request.
